# Age of acquisition and naming performance in Frisian-Dutch bilingual speakers with dementia

**DOI:** 10.1590/S1980-57642014DN83000009

**Published:** 2014

**Authors:** Wencke S. Veenstra, Mark Huisman, Nick Miller

**Affiliations:** 1 University of Groningen, University Medical Center Groningen, Department of Neurology and Department of Neurosurgery, Groningen, The Netherlands; 2 University of Groningen, Department of Sociology, Groningen, The Netherlands; 3 Speech and Language Sciences, University of Newcastle, Great Britain

**Keywords:** age of acquisition, bilingualism, picture naming, dementia

## Abstract

Age of acquisition (AoA) of words is a recognised variable affecting language processing in speakers with and without language disorders. For bi- and multilingual speakers their languages can be differentially affected in neurological illness. Study of language loss in bilingual speakers with dementia has been relatively neglected.

**Objective:**

We investigated whether AoA of words was associated with level of naming impairment in bilingual speakers with probable Alzheimer's dementia within and across their languages.

**Methods:**

Twenty-six Frisian-Dutch bilinguals with mild to moderate dementia named 90 pictures in each language, employing items with rated AoA and other word variable measures matched across languages. Quantitative (totals correct) and qualitative (error types and (in)appropriate switching) aspects were measured.

**Results:**

Impaired retrieval occurred in Frisian (Language 1) and Dutch (Language 2), with a significant effect of AoA on naming in both languages. Earlier acquired words were better preserved and retrieved. Performance was identical across languages, but better in Dutch when controlling for covariates. However, participants demonstrated more inappropriate code switching within the Frisian test setting. On qualitative analysis, no differences in overall error distribution were found between languages for early or late acquired words. There existed a significantly higher percentage of semantically than visually-related errors.

**Conclusion:**

These findings have implications for understanding problems in lexical retrieval among bilingual individuals with dementia and its relation to decline in other cognitive functions which may play a role in inappropriate code switching. We discuss the findings in the light of the close relationship between Frisian and Dutch and the pattern of usage across the life-span.

## Abstract (pt)

A idade de aquisição (IA) de palavras é uma variável reconhecida que interfere no processamento da linguagem em falantes com e sem distúrbios de linguagem. Para falantes bilíngues e multilíngues, a linguagem pode ser afetada diferenciadamente nas doenças neurológicas. O estudo da deterioração de linguagem em indivíduos falantes bilíngues com demência tem sido relativamente negligenciado.

**Objetivo:**

Nós investigamos se a IA de palavras estava associada ao grau de comprometimento da nomeação em indivíduos bilíngues com demência de Alzheimer provável em uma língua e entre as línguas faladas por eles.

**Métodos:**

Trinta e seis bilíngues Frisio-holandeses com demência de grau leve a moderado nomearam 90 figuras em cada uma das línguas, pareadas quanto a IA e outras variáveis. Aspectos quantitativos (numero de acertos) e qualitativos (tipos de erros e trocas inadequadas de código) foram mensurados.

**Resultados:**

Déficits de resgate lexical ocorreram no Frisio (Língua 1) e Holandês (Língua 2) com efeito significante da IA na nomeação em ambas as línguas. Palavras adquiridas mais precocemente foram mais preservadas e melhor resgatadas. O desempenho foi idêntico entre as línguas, mas melhor no Holandês, quando as covariáveis foram controladas. Entretanto, os participantes demonstraram mais mudanças impróprias de código no contexto do teste em Frísio. Na análise qualitativa não houve diferenças na distribuição global dos erros entre as línguas tanto para as palavras adquiridas precoce ou tardiamente. Houve uma porcentagem significativamente mais alta de erros semanticamente relacionados em relação aos visualmente relacionados.

**Conclusão:**

Os achados tem implicações para a compreensão de distúrbios de resgate lexical em indivíduos bilíngues com demência e a sua relação com o declínio em outras funções cognitivas que pode desempenhar um papel na inadequada mudança de código. Nós discutimos os achados à luz da estreita relação entre Frisio e Holandês e o padrão de uso durante a vida.

## INTRODUCTION

This study concerns the effects of age of acquisition (AoA) of a language and words within that language on naming performance in bilingual speakers with dementia. Studying language acquisition and attrition in healthy bilingual speakers has offered important insights into the development and processing of language in the brain, not just for bilingual speakers, but also for monolingual subjects.^1-5^ Study of breakdown across languages in people with neurological impairment has complemented these studies to further enrich the field in areas such as representation and processing of different languages or subsystems (e.g. phonology, lexicon, morphology) in the brain and the influence of psycholinguistic factors on outcomes in neurorehabilitation or decline.^6-11^ Sociolinguistic-oriented studies have emphasized the importance of social-psychological factors in language maintenance and loss across the lifespan.^2,3,12^ Differentiation of usage has been implicated as a variable affecting performance and outcomes – for instance role of a language as the medium for education and administration versus for daily living; or variation in the place and interlocutor of usage (e.g. at work vs at home; with spouse and friends vs with strangers). The importance of such variables is also that they in turn may influence relative levels of activation and inhibition and thereby determine ease of access to a particular language at a given time and in a given place.^5,13-15^

AoA (age of acquisition) of a language or of words within a language has been one of the variables that has received increasing attention as a factor that influences performance in mono- and bilingual speakers and may explain the relative vulnerability of their different languages to disruption from normal ageing processes or neurological disorders. AoA has been demonstrated to influence a range of linguistic functions and processes including word naming, recognition accuracy, reaction times on lexical decision and retention and attrition.^8,15-23^ Catling et al.,^24^ found such an effect in neurologically healthy subjects even after short controlled (frequency x order of acquisition) exposure to a computer delivered artificial language learning task.

As becomes apparent later, how one determines what counts as early vs later acquisition, the relationship of words across languages (in particular non-cognate vs cognate) and the relative dominance between language pairs can be important. For example, Cuetos et al.,^25^ studied the influence of frequency and AoA of words in English and Spanish individuals with Alzheimer's. The authors found AoA estimates based on adult (as opposed to younger person) ratings of AoA strongly predicted naming behaviour, as did word frequency derived from estimates based on older written texts (in contrast to recent ratings derived from film subtitles). When adult frequency estimates were replaced by estimates based on lifetime frequency of usage this did not lessen the effects of AoA.

Costa et al.,^26^ investigated naming in 71 Catalan-Spanish bilingual patients – 24 with mild cognitive impairment and 47 with Alzheimer's and found parallel deterioration in the speakers with Alzheimer's. For all groups, cognate status of words and word frequency interacted with AoA as determinants of performance, as found previously.^27^ However, Verreyt et al.^28^ suggests that the effect may only be operative when both languages are potentially activated.

Gollan et al.,^29^ examined naming in relation to the dominant or nondominant status of the languages of participants with dementia (Spanish-English). Against expectations, that the person's non-dominant language would be more vulnerable, they found that the dominant language could be more susceptible to disruption. Their explanation for this was that Alzheimer's affects richer semantic representations and therefore might be manifest earlier in the more proficient language. These brief illustrations underline the activeness of debates in the field and the necessity for further investigation.

Several theoretical accounts of word retrieval have considered that word frequency represents an important determinant of the speed and accuracy with which a word is recognized and responded to.^30,31^ High-frequency words are recognized more rapidly and more accurately than low-frequency words. Re-analysing data from Oldfield and Wingfield, Morrison et al.^32^ found AoA to be a significant determinant of object naming speed and that the relationship of word frequency to performance is significantly diminished or removed once its correlation with other variables, such as AoA and word length, is taken into account. This concurs with older findings.^33,34^ Of course, high-frequency words are typically learned earlier in life and tend to be short. Thus, it is possible that previous reports of frequency effects in word naming and/or word length in word naming may in fact have been AoA effects. Morrison et al.,^35^ showed a marked effect of rated AoA in naming matched on frequency, but no frequency effect in naming matched on rated AoA, indicating AoA rather than frequency is the major determinant of naming speed. The strength of support therefore appears to suggest that, all other variables being equal, words learned earlier in life are retrieved faster than later acquired words,^36,37^ and early acquired words may be more resistant to the effects of some forms of brain injury than later acquired words.^38,39^ We aimed to ascertain whether this pertained in bilingual speakers and whether the difference in AoA of their languages and words within those languages would confer some immunity to degradation of naming in earlier acquired words and languages in the face of dementia.

Further examination of this question will help confirm or refute findings concerning the effects of AoA on lexical performance. It can also afford insights into understanding the consequences of such effects in judging clinical prognosis in language attrition in bilingual speakers with dementia. Accordingly, in this cross-sectional study, we aimed to assess picture naming performance in Frisian-Dutch sequential bilingual speakers with dementia who were highly proficient in both languages before the onset of decline. We hypothesized that within languages, early acquired words would be more resistant to the effects of dementia than later acquired words, and that between languages Dutch (as the later acquired language) would be more susceptible to impairment.

## METHODS

**Participants.** We recruited bilingual Frisian and Dutch residents from a large psycho-geriatric nursing home in Fryslân. Inclusion was based on medical histories and the presence of cognitive impairments confirmed by neuropsychological tests, including memory, orientation, insight and word finding difficulties. The diagnosis of probable Alzheimer's disease (AD) was made according to the NINCDS-ADRDA criteria. The study was carried out following the principles of the declaration of Helsinki (http://www.wma.net/en/30publications/10policies/b3/) on conduct of medical research. Agreement to recruit from the nursing home was granted by the medical director with agreement of the staff. Potential participants and their families received full information about the study and their role. Participation was only after informed, voluntary consent, from both carers and the person with dementia.

Twenty-six patients with probable AD participated in the study (21 female), with a median age of 86 years (IQR 81-89, range 61-96 years). Time since diagnosis of dementia ranged from 1.5-10 years, with a median of 4.5 (IQR 2.25-9.0). Time in formal education ranged from 3-16 years (median 6, IQR 6-8 years), the majority having completed only elementary school.

Prior to dementia onset all participants had been highly proficiency bilinguals as determined by the History of Bilingualism Questionnaire.^40^ They acquired Frisian as their native language from birth. Two had first been exposed to Dutch at age 3 years, the rest at age 6 years on school entry. Subsequently they used both in an environment where Frisian and Dutch were used alternately. All used Dutch on a regular basis prior to dementia onset. However, in all cases, Frisian was considered the most dominant language throughout their lives. At the time of testing, eight participants considered themselves equally proficient in both languages, the rest dominant in Frisian. Four had not learned to write Dutch, thirteen did not write Frisian. Two did not read Frisian, but all read Dutch.

**Procedures.** Participants named a set of 90 black-and-white hard copy drawings of objects (Appendix 1), selected from the 391 items, all nouns, of the European Naming Test.^41^ Two equivalent lists were constructed for each language, which included cognates. The following word variables were recorded.

*Name agreement,* the extent to which subjects agree on the target name for a picture, was available for Dutch norms for all original 391 pictured items, based on two groups of healthy males and females, aged 60-75 with either less than 10 years, or more than 10 years formal education. Frisian measures of name agreement were obtained as part of the current work from six healthy bilingual speakers of Frisian and Dutch who were asked to name all the initial 391 pictures in both languages. The final selection of pictures that were used in this study comprised the 90 stimuli with values for Dutch name agreement median of 93% (IQR 88-98, range 78-100%) and for Frisian median of 100% (IQR 80-100, range 70-100%).

*Word frequency* for Dutch was taken from the CELEX database.^42^ Frisian word frequencies were obtained from the Frisian word count database of the Fryske Akademy, the main research institute for Frisian language and history.

*Age of acquisition.* Ideally, word learning age should be derived from a child language database. In the absence of such a database, researchers have relied on adult estimated AoA ratings. AoA ratings correlate highly with objective ratings of word learning age. A high correlation was obtained between rated AoA and the rank order of the words in the norms on the standardized Chrichton and the Mill Hill Vocabulary Scales.^33,34^ Morrison et al.,^43^ showed a significant correlation between children's naming performance and adult AoA ratings for 297 pictorial nouns, concluding that rated word learning age is both a reliable and a valid measure of AoA.

We obtained AoA norms using a scale with seven age bands beginning with 0-2 years and increasing 2 years at a time to 11-12 years of age, with the oldest response being 13 years or older.^44^ Ratings were collected from 17 healthy, highly proficient bilingual adults (11 females), with a median age of 29 years (range 18-62 years). All were native speakers of Frisian, and their second language was Dutch. They were presented with the 90 Frisian target words and their Dutch translation equivalents and rated when they believed they had learned each word, in either spoken or written form. AoA ratings were averaged across the 17 raters, and the mean score for the items calculated for both languages (Appendix 1). Following Morrison et al and others we used a cut-off of mean of 8 years to distinguish early from late acquired words.

*Word length* was defined as the number of phonemes for both Frisian and Dutch names. Mean length for Frisian was 6.2 (SD 2.8; range 2-13) and for Dutch was 5.75 (SD 2.5, range 2-12).

**Testing.** Participants were seen on separate occasions for testing in Frisian and Dutch, within an interval of three to seven days. Order was randomised across participants. Each participant received instruction using practice trials. Testing commenced once comprehension of the task was confirmed.

Participants were asked to name the picture. If they failed to respond or indicated difficulty perceiving the meaning of the picture, a semantic cue was provided. If they were still unable to name the picture, a phonemic cue was given, the first sound of the target word. These contrasting cues were employed to permit insights into whether misnaming might be due to misidentification or degradation of knowledge of the concept.

Testing was conducted by two different examiners, one a monolingual Dutch speaker, the other a bilingual Frisian-Dutch speaker. On both occasions, the sessions were entirely monolingual, including all conversation, instructions and administration of the test. The monolingual examiner could easily claim she did not understand when spoken to in Frisian. If Frisian was used she asked for the Dutch translation. However, the bilingual examiner could not feign ignorance of Dutch since no one is monolingual in Frisian. She maintained Frisian throughout. If the individual switched to Dutch she repeated in Frisian what had been said and continued in Frisian. This represented a slight inconsistency between languages. However, there were very few instances of this situation (see results).

Responses were scored independently by two raters. Correct responses and immediately self-corrected responses counted as correct. Correct responses after semantic or phonemic cuing were totalled separately. Errors were classified as incorrect when a speaker gave no response, a wrong name, or the translation equivalent of a target word (i.e. there was inappropriate code switching). Also noted, was whether code switching occurred after cued items. Incorrect responses were further divided into related or unrelated to the target word. Related responses were categorised according to visual or linguistic (semantic; phonological) similarity to the target item. Unrelated responses were subdivided into perseverations and no response.

## RESULTS

As in previous studies there existed a significant correlation between AoA and frequency overall (Pearson's r –.503, p=<0.001) as well as within the separate languages (Frisian r –.577; Dutch r –.614), with later acquired words tending to be lower frequency. AoA and word length also correlated significantly (overall r .340, p=<0.01; Frisian r .523; Dutch r .543), with longer words being later acquired. Name agreement did not correlate significantly with AoA, frequency or word length.

**Naming performance.** Table 1 summarises the correct and self-corrected responses for all items. Table 2 separates correct responses according to early versus late acquired words.

**Table 1:** Mean number of raw score (n max 90) correct and self-corrected responses (with standard deviations and 95% C.I. - confidence intervals) per target word in Frisian and Dutch.

	Correct responses			Self-corrected responses	
	Mean (SD)	95% CI		Mean	95% CI
Frisian	11.61 (6.80)	[10.19, 13.04]		1.26 (1.40)	[0.96, 1.55]
Dutch	11.68 (5.38)	[10.55, 12.80]		0.89 (1.02)	[0.67, 1.10]

**Table 2:** Correct responses separated according to AoA.

	Early AoA				Late AoA		
	n	Mean (SD)	95% CI		n	Mean	95% CI
Frisian	72	14.8 (6.3)	[13.3, 16.3]		18	5.1 (5.7)	[2.2, 7.9]
Dutch	38	16.1 (3.5)	[15.0, 17.3]		52	10.0 (5.2)	[8.5, 11.4]

There was almost identical mean total correct responses in the two languages (Table 1), with a nonsignificant higher percentage of self-corrections in Frisian. A repeated-measures analysis of covariance was performed on the set of 90 pictured items to test the effect of language on correct plus self-corrected responses. The covariates included were AoA, word frequency and word length. With these variables controlled, the analysis showed a significant effect of language [*F*(1,86)=10.76, *p*=0.001], with more correct responses in Dutch (Table 2); and a significant effect of AoA [*F*(1,86)=24.37, *p* < 0.001] on the number of correct responses in both Frisian and Dutch (Table 2). Earlier acquired words were more likely to be correct. The mean number of correct responses, adjusted for the influence of the covariates, was 10.76 [99% C.I 9.04, 12.48] for Frisian and 14.68 [99% C.I. 12.96, 16.36] for Dutch.

**Code switching.** A further repeated-measures analysis of covariance was performed to test the effect of language on code switching (naming the item correctly but in the other language). The covariates included were AoA, word frequency, and word length. There was a significant effect of language [*F*(1,86)=13.42, *p* < 0.001] and AoA [*F*(1,86)=17.73, *p* < 0.001] on the number of other-language responses. More other language intrusions were observed within the Frisian setting, i.e. intrusion of Dutch into Frisian (corrected mean 6.15 [CI 4.49, 7.81]) than within the Dutch setting (corrected mean 1.94 [CI 0.28, 3.59]), i.e. intrusion of Frisian into Dutch. No difference was found within the Dutch data between early and late acquired words, but in Frisian a higher amount of code-switching was observed in earlier compared to later acquired words.

**Errors.** The proportion of error types, as outlined in the methods section, and the average number of errors per target word appear in Table 3. No difference in overall error distribution was found between languages, either for early or late acquired words. The results indicate a significantly higher percentage of semantically than visually related errors in both languages.

**Table 3:** Classification and distribution of errors across Frisian and Dutch for early and late AoA.

Error type	Frisian					Dutch			
	Translated examples	n	mean	%		Translated examples	n	mean	%
Visual	Waterfall → Shawl	90	1.18	4.5		Eye ® Football	90	1.09	4.2
Semantic	Elephant → Cow	90	4.52	17.4		Pear → Banana	90	5.06	19.4
Phonological	Dog/hun/ → /huz/	90	0.16	0.6		Axe /bil/ → /vil/	90	0.12	0.5
Unrelated	Forehead → Dress	90	0.87	3.3		Tie → Stool	90	0.91	3.5
Perseveration		90	0.07	0.3			90	0.04	0.2
No response		90	0.63	2.4			90	0.84	3.2
Early		72	5.75	22.1			38	4.63	17.8
Late		18	14.11	54.3			52	10.58	40.7

## DISCUSSION

Our results confirm previous reports of deficits in word finding in dementia. Consistent with other investigations of naming abilities in bilingual speakers with dementia, we found a similar severity and nature of problems in lexical retrieval in both the first and second acquired language, supporting the contention that the neurophysiological and neuropsychological processes underlying speech output are shared between the languages of a bilingual person.^10,26,29,45^ Where apparent differences between the languages did emerge these may be interpreted (below) within a framework where explanations relate to patterns of usage and relative proficiency in different domains and situations that may influence levels of activation, rather than differential neural representation per se.^14,15,26,45-47^

We hypothesized that naming accuracy would be greater in an earlier acquired language and that within languages, earlier acquired words would be less susceptible to error in people with dementia. Previous work, as cited above, has suggested that less frequent and longer words would be more poorly named, and that object names with high name agreement would be retrieved faster and more accurately than object names with lower name agreement; however, once these variables were controlled for, AoA would emerge as a sole predictor. The present results confirm effects for frequency and length, but crucially also indicate that when words are controlled for these variables, there remains a significant effect of AoA on naming within languages. Name agreement did not feature as a significant variable once other variables were factored out.

However, the hypothesis that Dutch, the later acquired language, would be more impaired than Frisian, was not borne out. Further, the current group demonstrated more inappropriate code switching within the first language (Frisian) setting than Dutch, despite always choosing the appropriate language in conversation.

Such findings reflect those of others. De Santi et al.,^48^ reported that their bilingual patient with dementia always chose the appropriate language in conversation but demonstrated code-switching problems in the first language. They explained this pattern by the fact that the patient had learned both languages almost simultaneously. This does not apply to the current speakers where there was a distinct separation in acquisition of their two languages.

Hyltenstam et al.,^49^ investigated two premorbidly highly fluent bilingual speakers with Alzheimer's. Their late bilingual was more likely to employ his first language. Whilst the early bilingual showed no tendency to overuse one language compared to the other, she did not always choose the appropriate one. They speculated that the early bilingual suffered from a language choice problem and the late bilingual from a language separation problem, a disability in inhibiting the native language while producing the second language. The basis, and usefulness of the distinction between choice and separation has been questioned due to its lack of theoretical validity and lack of practical feasibility in establishing the distinction.^50^ A more plausible suggestion for Hyltenstam et al's findings is that the underlying impairment for both was one of activation and inhibition, but that (at the time of testing) for their late bilingual there was stronger inhibition of the second language.^15^ This would also be in agreement with Heidlmayr et al.^14^ who investigated effects of frequency of use of a language on a Stroop colour task among neurologically intact highly proficient sequential bilinguals. Their findings supported the notion that top-down active inhibition of languages plays an important role in performance.

In the present study, in terms of relative activation-inhibition of languages in a situation, the findings may have arisen in the following fashion. Every effort was made to assure testing sessions were viewed as monolingual exchanges. Whilst speakers throughout their lives would have been used to encounters where interlocutors spoke no Frisian, there would have been next to none where the assumption was that the conversation partner understood no Dutch. The greater intrusion of Dutch naming into the Frisian setting here, which was perceived as formal, with a non-family member or close friend, may therefore reflect an underlying language activation readiness^14,15,51^ where in speaking Frisian with a stranger Dutch is not completely supressed, but in speaking Dutch the Frisian language may be. Further, whenever lexical retrieval difficulties arose in Frisian, a life-long accepted strategy would have been to code switch to Dutch, but the reverse would not be the case. These may all have added to the tendency here for Dutch, the later acquired language, to intrude into Frisian conversations but not vice versa.

Certain contextual features of this study might also colour the understanding of the data, in particular interpreting the relationship of AoA effects across languages. Although speakers perceive Frisian and Dutch as separate languages, structurally and phonologically they are extremely close and thus this study is similar to ones employing bilingual speakers of e.g. Spanish-Catalan, Spanish-Galician or Friulian-Italian, Veneto-Italian. For several items in this study, the difference was more one of pronunciation rather than radically different lexical item (e.g. Frisian *kaam* Dutch *kam* (comb)). For these words, the Dutch version was indeed acquired later, but its closeness to the Frisian form meant the nature of its representation and processing may be different to words that contrast completely (e.g. Frisian *kaai*, Dutch *sleutel* (key)). Studies demonstrate that cross-language cognates behave differently in terms of frequency and AoA effects compared to non-cognate lexical items.^26,27,52^ In effect, AoA for cognate pairs should be taken from the AoA in the first learned language; frequency is better represented by a cumulative cross-language measure rather than separate measures for each language.

The cognitive consequences of the presence of a significant number of cognates may therefore have somewhat biased the Dutch AoA outcomes here. However, with regards to performance on cognates, Verreyt et al.^28^ showed in a single case study of a bilingual aphasic speaker, that cognate effects may be less straightforward than simply they do or do not behave differently to non-cognates. The authors found an effect of cognate status on naming in tasks where both languages could be expected to be activated, but almost no effect on tasks where languages were selectively targeted. Thus further investigation of cognate effects is warranted.

Pattern of usage across the life-span may also represent a factor that influenced results. Frisian was acquired from birth. As the participants grew up (before the advent of television and widespread radio) Frisian was likely to be the sole language heard before school entry. From school years onwards the speakers employed Frisian and Dutch in a balanced and highly proficient fashion. They heard, read and spoke both languages extensively. However, there may have been subtle variations in balance that had consequences for test scores here. Dutch became the language for more formal discourse, reading, writing and later national TV and radio entertainment (but with Frisian regional stations). Literacy in a language also influences phonological organisation and output.^53^

This may have introduced a bias for later acquired Dutch words to be spoken more frequently than later acquired Frisian words.^46^ The interpretation is not supported by Cuetos et al.^54^ who tested the claim that words acquired later in life would show a greater frequency (as opposed to AoA) effect. They in fact found the reverse, suggesting that amount of exposure across the whole life-span may interact with AoA. We did not conduct fine-grained language dominance tests with participants. The assumption was that, similar to unaffected individuals, their usage of Dutch and Frisian was equal in distribution, frequency and proficiency. This underlines the importance in future studies of gathering data not just on overall usage of a language, but also on the domains of usage, in order to exclude this potential distortion.

Language dominance has been a factor proposed to affect performance and decline. The dominant language is considered easier to access and activate, especially given that dementia compromises executive function and top-down control processes. Gollan et al.,^29^ investigated the hypothesis that bilingual patients with dementia regress to using primarily their dominant language. Nevertheless, they found speakers with patterns of naming better or equal in their non-dominant language compared to dominant one, suggesting a speaker's non-dominant language is not necessarily differentially susceptible to disruption. They speculated that this may relate to the possibility that decline in dementia is first manifest in words with richer semantic networks, which would be the case for the dominant language.

Another possibility for why performance in some bilingual speakers may be poorer in their notionally dominant or first language than in the assumed non-dominant later language is where the former has been infrequently employed in later years and so may be already in a more advance state of attrition.^2,55^ In the current study, where participants had maintained both languages equally throughout their lives, this should not have been the case. Further, one should guard against assuming that an earlier acquired language is automatically the more dominant one. Dominance may well be a function of order and age of acquisition but is also determined by a host of other social and psychological variables pertaining to patterns of usage across the life-span.^2,5,12^

Turning to the pattern of errors shown by participants, semantic rather than visual features dominated. This supports the contention that (at least in mild to moderate stages of dementia) naming is predominantly compromised by degradation of semantic representations whilst visual object analysis is not influenced by AoA.^10,21,25,29^ Alternative accounts for the profile could be that AoA may primarily affect processes that occur prior to others more sensitive to word frequency or object recognition,^56,57^ and/or earlier acquired words are produced faster because the corresponding concepts have richer sensory-visual features acquired through direct sensory experience.^22^ Whether semantic or visual errors predominate may be steered by the nature of the task, for instance naming versus object decision.^21^ Further investigations comparing tasks and elicitation procedures and tracking evolution of error patterns longitudinally in bilingual speakers^11,45^ similar to studies conducted with monolingual speakers^10,11^ are still required.

As regards potential clinical lessons, the present findings confirm that AoA significantly influences naming performance in bilingual speakers with dementia. This should be taken into consideration when devising and applying assessments for this population. The outcomes also suggest taking AoA into account when searching for words or devising materials to support information giving and rehabilitation of bilingual people with dementia. Interpretation of the results further suggests it is important to gather information not just on overall pattern of acquisition and usage of the languages but to focus on more subtle aspects of usage or exposure and elements in interactions that can affect levels of activation of the language overall or specific domains of vocabulary within the different languages. There may be a role for exploiting AoA in picture naming as a variable sensitive to cognitive decline and detection of cognitive changes.^8,39^

Firmer confirmation of these suppositions is awaited through replication of the study with more distantly or unrelated language pairs. Future studies can also in form which aspects of languages (distance, cognates) influence inhibitory control and error monitoring (as part of executive dysfunction) in bilingual speakers with neurological diseases. Finally, it is clear that we do not yet fully understand the nature of the origin or effects of AoA. The effect may not be specifically linguistic or more narrowly lexical but a manifestation of a more general property of order of acquisition.^8,39^

## APPENDIX 1 Picture names in Frisian and Dutch with mean rated age of acquisition (AoA) and word frequency.

**Table:**

Frisian (L1)	Dutch (L2)	English	L1 AoA	L2 AoA	L1 FREQ	L2 FREQ
Strykizer	Strijkijzer	Iron	4.3	8.2	–0.075	0
Kroade/kroaie	Kruiwagen	Wheelbarrow	3.6	8.1	1.393	0.699
Kaam	Kam	Comb	4.1	6.1	0.721	0.903
Flesse	Fles	Bottle	3.2	6.5	1.488	2.049
Earrebarre	Ooievaar	Stork	9.5	8.1	0.808	0.301
Draak	Vlieger	Kite	9	7.2	1.020	0.778
Hûn	Hond	Dog	2.6	6.8	2.071	2.225
Swimbad	Zwembad	Swimming pool	5.1	8.1	0.841	1.176
Geit	Geit	Goat	2.8	7	1.227	0.954
Spjocht	Specht	Woodpecker	10.1	8.8	0.071	0.301
Toskboarstel	Tandenborstel	Toothbrush	3.7	7.4	–0.121	0.602
Skermer	Schermer	Fencer	10.3	10.5	–1.075	0
Tsjerke	Kerk	Church	4.4	7.5	2.527	2.312
Matroas	Matroos	Sailor	6.4	8.2	0.553	1.146
Poddestuollen	Paddestoel	Mushroom	4.6	7.5	0.537	0.954
Foarholle	Voorhoofd	Forehead	4.9	8.1	1.561	1.724
Seage/sage	Zaag	Saw	5.1	7.8	0.940	0.477
Kopke	Kopje	Cup	2.8	6.9	1.670	1.672
Leadjitter	Loodgieter	Plumber	9.2	10.1	–0.598	0.301
Finster/rút	Raam	Window(frame)	3.6	7	2.073	2.241
Pylk	Pijl	Arrow	6.4	8.1	0.948	1.204
Hân	Hand	Hand	2.5	6.8	2.764	3.012
Mûs	Muis	Mouse	2.9	7.1	0.940	1.322
Biezem	Bezem	Broom	4.4	7.1	0.838	0.602
Rêchtas	Rugzak	Rucksack	8.1	8.6	–0.015	0.903
Bile	Bijl	Axe	5.9	8.4	1.149	1.041
Panne	Pan	Pan	3.6	7	1.467	1.544
Fioele	Viool	Violin	8.4	9	1.163	1.079
Bûslantearne	Zaklantaarn	Torch	8.8	8.2	0.568	0.602
Blomkoal	Bloemkool	Cauliflower	4.6	8.1	0.462	0.301
Fluittsjettel	Fluitketel	Kettle	6.3	9	–0.531	0
Noas	Neus	Nose	2.2	7	1.920	2.004
Potlead/poatlead	Potlood	Pencil	3.6	7	0.976	1.079
Stryk/strik	Stropdas	Tie	6.1	9.1	0.469	0.778
(Foar)doar	Deur	Door	3	7	2.714	2.575
Hammer	Hamer	Hammer	4.1	8.1	1.066	1.041
Fierrekiker	Verrekijker	Binoculars	8.4	9.1	0.115	0.778
Trep	Trap(je)	Stairs	3.5	7.2	1.542	2.065
Stofsûger	Stofzuiger	Vacuum cleaner	5	8.9	–0.015	0.477
Fuotballer	Voetballer	Footballer	5.1	8.3	0.086	0.602
Skjirre	Schaar	Scissors	4.1	7.2	1.055	0.845
Leppel	Lepel	Spoon	2.9	7.2	1.042	1.255
Ule	Uil	Owl	8.1	8.8	0.805	0.903
Mitselder	Metselaar	Mason	9.2	9.7	–0.146	0.477
Bosk	Bos	Woods	4.4	7.8	1.997	1.924
K(j)ers/kears	Kaars	Candle	4.6	7.2	1.305	1.362
Tromme	Trommel	Drum	5.4	7	0.911	1.301
Skroevedraaier	Schroevendraaier	Screwdriver	8.2	8.6	–0.015	0.301
Kroan	Kroon	Crown	6.3	9.1	1.257	1.415
Garaazje	Garage	Garage	5.5	8.1	0.931	1.176
Hynder	Paard	Horse	3	7.1	2.117	2.199
Sikehûs	Ziekenhuis	Hospital	5.2	8.4	1.541	1.973
Slide	Slee	Sledge	3.7	7.4	0.811	0.301
Flear(e)mûs	Vleermuis	Bat	9	9.6	0.409	0.778
Skuonmakker	Schoenmaker	Cobbler	7.5	8.4	0.895	0.602
Each	Oog	Eye	2.5	6.8	2.312	2.914
Ein(fûgel)	Eend	Duck	3.1	7	0.323	1.38
Roas	Roos	Rose	5.4	8.2	1.150	1.462
Oaljefant	Olifant	Elephant	6.6	8.8	0.583	1
Ierdbeien	Aardbeien	Strawberries	4.9	7	–0.172	0.699
Fleanmasine	Vliegtuig	Aeroplane	8.2	9.1	0.286	1.716
Skimerlampe	Schemerlamp	Lamp	6.9	8.6	0.305	0.477
Skuon	Schoenen	Shoes	3.1	8.4	1.566	1.833
Hin	Kip	Chicken	2.9	7.1	1.347	1.519
Ferkearsp(o)lysje	Verkeersagent	Traffic police	9	9.5	–0.677	0
Foet	Voet	Foot	2.6	7	1.994	2.352
Swan	Zwaan	Swan	4.8	7	0.800	0.903
Kaai	Sleutel	Key	5.8	8.1	1.439	1.69
Pankoekspanne	Koekenpan	Frying pan	6.1	8.2	0.129	0
Skiep	Schaap	Sheep	2.9	7.1	1.868	1.415
Amer	Emmer	Bucket	3.7	8.1	1.335	1.342
Skyldpod	Schildpad	Turtle	6.5	8.8	–0.376	0.778
Skearmeske	Scheermes	Razor	8.5	9.2	0.247	0.477
Knyptange	Nijptang	Pliers	7.1	8.9	0.129	0
Koaningin(ne)	Koningin	Queen	7.6	9.2	1.512	1.613
Liuw	Leeuw	Lion	6.3	9.1	1.177	1.362
Glês	Glas	Glass	3.9	8.3	1.775	2.185
Ear	Oor	Ear	2.4	7	1.856	2.041
Gieter	Gieter	Watering can	5.2	8.6	–0.899	0
Hobbelhynder	Hobbelpaard	Rocking horse	3.1	8.3	–0.335	0
Par	Peer	Pear	4.8	7	0.846	1
Ier(d)appels	Aardappels	Potatoes	4.4	8.1	1.727	1.447
Ko	Koe	Cow	2.4	7.4	1.313	1.556
Strykplanke	Strijkplank	Ironingboard	6.2	9.4	–0.598	0
Hûs	Huis	House	2.4	7	2.986	2.799
Sinneblom	Zonnebloem	Sunflower	7.1	8.8	–0.075	0.301
Wetterfal	Waterval	Waterfall	9.1	10.2	0.086	0.903
Skoarstien	Schoorsteen	Chimney	6.5	9.2	1.203	1.041
Doas/doaze	Doos	Box	4.5	7.2	1.212	1.591
Fjoertoer	Vuurtoren	Lighthouse	8.4	9.6	0.039	0.477
Mean (SD)			5.38 (2.21)	8.04 (0.96)	0.88 (0.86)	1.12 (0.74)
